# Global contribution of pelagic fungi to protein degradation in the ocean

**DOI:** 10.1186/s40168-022-01329-5

**Published:** 2022-09-01

**Authors:** Eva Breyer, Zihao Zhao, Gerhard J. Herndl, Federico Baltar

**Affiliations:** 1 Department of Functional and Evolutionary Ecology, University of Vienna, Djerassiplatz 1, 1030 Vienna, Austria; 2 NIOZ, Department of Marine Microbiology and Biogeochemistry, Royal Netherlands Institute for Sea Research, Utrecht University, AB Den Burg, The Netherlands; 3 Vienna Metabolomics Center, University of Vienna, Djerassiplatz 1, 1030 Vienna, Austria

**Keywords:** Pelagic fungi, Proteases, Metagenomics, Metatranscriptomics, Global ocean, Nitrogen cycle

## Abstract

**Background:**

Fungi are important degraders of organic matter responsible for reintegration of nutrients into global food chains in freshwater and soil environments. Recent evidence suggests that they are ubiquitously present in the oceanic water column where they play an active role in the degradation of carbohydrates. However, their role in processing other abundant biomolecules in the ocean in comparison with that of prokaryotes remains enigmatic. Here, we performed a global-ocean multi-omics analysis of all fungal-affiliated peptidases (main enzymes responsible for cleaving proteins), which constitute the major fraction (> 50%) of marine living and detrital biomass. We determined the abundance, expression, diversity, taxonomic affiliation, and functional classification of the genes encoding all pelagic fungal peptidases from the epi- and mesopelagic layers.

**Results:**

We found that pelagic fungi are active contributors to protein degradation and nitrogen cycling in the global ocean. Dothideomycetes are the main fungi responsible for protease activity in the surface layers, whereas Leotiomycetes dominate in the mesopelagic realm. Gene abundance, diversity, and expression increased with increasing depth, similar to fungal CAZymes. This contrasts with the total occurrence of prokaryotic peptidases and CAZymes which are more uniformly distributed in the oceanic water column, suggesting potentially different ecological niches of fungi and prokaryotes. In-depth analysis of the most widely expressed fungal protease revealed the potentially dominating role of saprotrophic nutrition in the oceans.

**Conclusions:**

Our findings expand the current knowledge on the role of oceanic fungi in the carbon cycle (carbohydrates) to the so far unknown global participation in nitrogen (proteins) degradation, highlighting potentially different ecological niches occupied by fungi and prokaryotes in the global ocean.

Video Abstract

**Supplementary Information:**

The online version contains supplementary material available at 10.1186/s40168-022-01329-5.

## Background

Microbes make up around 70% of the total marine biomass [1] and are involved in complex functional and phylogenetic networks with all three organismal domains of life and viruses [2]. They harbor a set of genes responsible for driving major redox reactions that are crucial for controlling the remineralization of organic material [3]. Most of the research on the role of microbes in the oceanic nutrient cycling has focused on prokaryotes. Little is known on the role of pelagic fungi in the cycling of organic matter in the ocean despite fungi being recognized as key elements in remineralizing nutrients and degrading organic matter in the terrestrial and freshwater environment [4]. However, recent studies revealed that pelagic fungi were found to dominate the microbial biomass in deep-sea marine snow [5] and exhibited biomass concentrations similar to that of prokaryotes during phytoplankton blooms [6]. Moreover, by infecting inedible phytoplankton, parasitic fungi are suggested to act as trophic bridge via the fungal shunt by producing zoospores that are consumed by zooplankton, a process defined as the “mycoloop” [7–9].

Recent evidence also indicates that pelagic fungi play a potentially important role in the marine carbon cycle [10–12]. A global-ocean scale multi-omics study reported a widespread and active role of fungi in degrading carbohydrates by studying the diversity and expression of carbohydrate-active enzymes (CAZymes) phylogenetically affiliated to fungi [12]. These authors found that the abundance and diversity of total and secretory fungal CAZymes increased with increasing depth, which contrasts the rather constant depth distribution of the abundance and diversity of prokaryotic CAZymes [13]. This might indicate differential ecological niches and roles of pelagic fungi and prokaryotes in the degradation of carbohydrates in the ocean [12]. Nevertheless, although carbohydrates are major components of macromolecules in marine organisms (ca.10%), proteins constitute the major fraction (> 50%) of the organic matter making up marine planktonic cells [14].

Thus, to obtain a more complete understanding of the role of pelagic fungi in the remineralization of organic matter, we performed a systematic investigation of the role of oceanic pelagic fungi in the degradation of proteins. We analyzed global metagenomic and metatranscriptomic data covering all major oceanic basins to reveal fungal peptidase activity in the epi- and mesopelagic waters. Also, the fungal taxa and peptidase classes responsible for protein degradation were determined. Based on the expression of CAZymes related to fungi, we hypothesized that fungi are also major contributors to the degradation of proteins in the oceanic water column. Moreover, we hypothesized that the same organisms dominating the expression of CAZymes might also dominate the expression of proteases. Finally, we hypothesized to find evidence of shared and distinct traits involved in the degradation of organic matter between pelagic fungi and prokaryotes. Our detailed analyses of fungal proteases allowed us to infer potential lifestyle strategies of oceanic mycoplankton. Also, the comparison of our results to recent similar efforts to characterize the global distribution of fungal-affiliated CAZymes [12] and of prokaryotic-affiliated CAZymes and proteases [13] allowed us to decipher potential similarities and differences in the role of fungi versus prokaryotes in the degradation of carbohydrates and proteins.

## Methods

The sequences and occurrences in the corresponding metagenomes and metatranscriptomes of marine eukaryotic genes were downloaded from literature [15]. For the metagenomics data, the accession number is PRJEB4352 and for the metatranscriptomics data PRJEB6609. Gene occurrence in the metagenomic and metatranscriptomic dataset is available at http://www.genoscope.cns.fr/tara/ “Tara Oceans eukaryote gene catalog.” Environmental parameters were downloaded from the original paper (Supplementary Data 5, Carradec et al. 2018). A Mantel test was performed between fungal peptidase beta diversity and environmental parameters to test the driving factors shaping fungal peptidase in marine environments. We used the presence of signal peptides as a proxy for secretory peptidase because signal peptides are short amino acid sequences in the amino terminus of proteins that direct proteins into or across membranes. Thus, the peptidase encoding genes containing signal peptide sequences can be probably translocated from the cytoplasmic to the periplasmic space as cell-associated extracellular enzymes “in contact to the outside” or transported to the outside of the cell as cell-free extracellular enzymes.

To identify peptidase-like sequences, the 116 million eukaryotic genes were first compared against the MEROPS database [16] (https://www.ebi.ac.uk/merops/) using DIAMOND version.0.8.36 blast [17] (*e*-value < 1 × 10^−20^). Sequences with positive hits were extracted for taxonomic identification using the lowest common ancestor algorithm adapted from DIAMOND v.0.8.36 [17] blast by searching against the NCBI nonredundant (NR, downloaded in March 2020) database. The top 10% hits with an *e*-value < 1 × 10^−5^ were used for taxonomic affiliation assessment (top 10). The functional annotations at the peptidase family level were further grouped into peptidase class level according to the common designations in the MEROPS database. SignalP [18] (5.0) was used to detect the presence of signal peptides for fungal sequences under the eukaryotic mode. Size fractions were defined as micro-mycobiome for samples from 0.8 to 5 μm (0.8–3 μm was used for samples from the mesopelagic waters as it was the only available range) and macro-mycobiome for samples from 5 to 2000 μm (3–2000 μm was used for samples from mesopelagic waters), consistent to our recent study on fungal CAZymes [12]. Data analysis was performed using R (R version 3.6.1, www.R-project.com). *Vegan* [19], *rtk* [20], and *ggplot2* [21] were used for ordination, diversity calculation, and visualization, respectively.

## Results and discussion

### Occurrence, secretory capacity, and α-diversity of fungal peptidases genes and transcripts in the global ocean

We examined the occurrence, diversity, functional classification, taxonomic affiliation, and metabolic expression of genes encoding proteases from 445 metagenomes and 440 metatranscriptomes from 68 Tara stations of size fractions ranging from 0.8 to 2000 μm, covering the epipelagic (0–200 m) and mesopelagic (200–1000 m) waters (see “Methods”). Of the 116 million nonredundant sequences of global ocean eukaryotic genes, 815,841 eukaryotic proteases sequences were retrieved. Fungi-affiliated sequences contributed 1.05% (8637 out of 815,841) to these eukaryotic protease sequences. The abundance of fungal protease sequences ranges from 1 to 3952 in the metagenomes and from 4 to 5601 in the metatranscriptomes. Ascomycota- (1 to 3681 in the metagenome, 1 to 5058 in the metatranscriptome) and Basidiomycota (1 to 269 in the metagenome, 1 to 528 in the metatranscriptome)-affiliated sequences showed highest count in both metagenomics and metatranscriptomic datasets, and sequences were predominantly assigned to serine peptidase (1 to 1789 in the metagenome, 1 to 1083 in the metatranscriptome) and metallopeptidase (1 to 1538 in the metagenome, 1 to 2393 in the metatranscriptome). The occurrence and expression values of all eukaryotic sequences in the metagenomic and metatranscriptomic dataset were downloaded from http://www.genoscope.cns.fr/tara/, and fungal-related values used in this analysis are subtracted and supplied as supplementary material (Supplementary Dataset S1).

The analysis of metagenomic (MetaG) and metatranscriptomic (MetaT) data of fungal peptidases using principal coordinate analysis (PCoA) revealed that oceanic fungal communities are clustered by water column depths into epipelagic (SRF, surface; MXL, mixed layer; DCM, deep chlorophyll maximum) and mesopelagic (MES) (Fig. S1). In contrast, size fractionation between micro-mycobiome (0.8–5 μm) and macro-mycobiome (5–2000 μm) exhibited no clear differentiation (Fig. S1). These results for pelagic fungal proteases (in both MetaG and MetaT datasets) are consistent with the patterns observed for fungal CAZymes, which exhibited a depth-dependent clustering but no clustering based on size [12]. A Mantel test between pelagic fungal peptidase profiles and environmental parameters showed that the fungal peptidases from the micro-fraction were significantly related to chlorophyll, phosphorus, and nitrogen at the metagenomics level but not to any environmental parameters at the metatranscriptomic level (Fig. S1). Analysis of the macro-fraction-affiliated fungal peptidases revealed that on the metagenome level, fungal peptidases were significantly related to temperature, oxygen, net primary production, and iron, whereas on the metatranscriptomic level, fungal peptidases were related to phosphorus, nitrogen, and iron. This suggests that pelagic fungal proteases are controlled by nutrients (particularly iron, phosphorus, and nitrogen) and organic matter availability (as indicated by net primary production and chlorophyll). Similar links between nutrients and organic matter availability were reported for pelagic fungal CAZyme genes and transcripts [12]. In contrast, the potential and expression of both peptidases and CAZymes affiliated to oceanic prokaryotes were linked to temperature, salinity, and oxygen [13]. This suggests that the cleaving of protein-rich substrates by pelagic fungi and prokaryotes is governed by different ecological factors as recently suggested for carbohydrates [12].

**Fig. 1:**
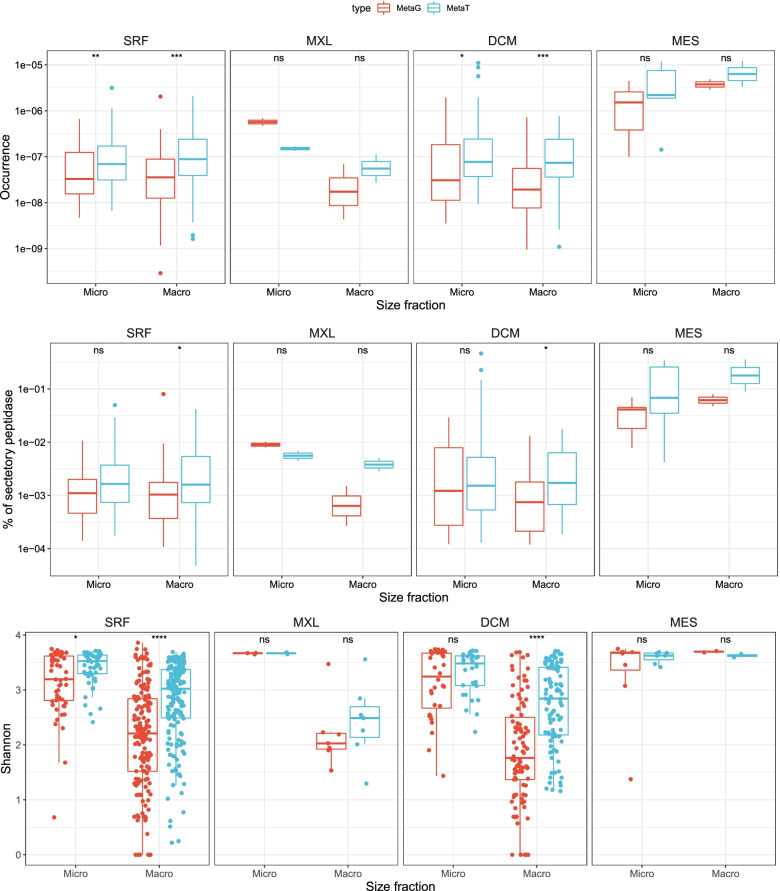
Occurrence (**A**), secretory capacity (**B**), and α-diversity (**C**) of genes and transcripts for fungal peptidases. Micro, micro-mycobiome (0.8–5 μm); macro, macro-mycobiome (5–2000 μm). Box shows median and interquartile range (IQR); whiskers show 1.5 × IQR of the lower and upper quartiles or range; outliers extend to the data range. Statistics are based on Wilcoxon test, *P < 0.05, **P < 0.01, ***P < 0.001, ****P < 0.0001, ns, not significant. SRF, surface; MXL, mixed layer; DCM, deep chlorophyll maximum; MES, mesopelagic

**Fig. 2:**
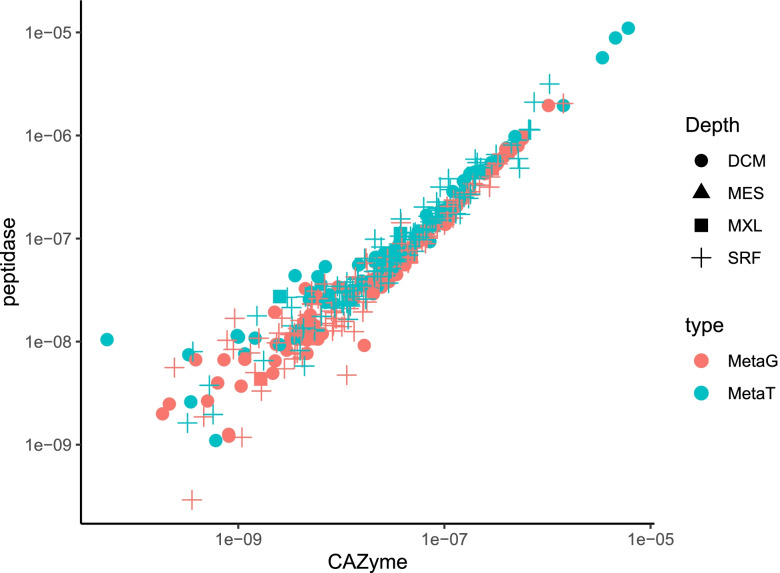
Correlation between fungal proteases genes and transcripts (from this study) with fungal CAZymes (from Zhao et al. [13]) in the global ocean epi- and mesopelagic waters. SRF, surface; MXL, mixed layer; DCM, deep chlorophyll maximum; MES, mesopelagic; MetaG, metagenomic; MetaT, metatranscriptomic

The ratio of transcripts to genes of both total (i.e., cytosol and secreted) and secretory peptidases was generally equal or higher than 1 throughout the water column, suggesting an active expression and secretion of fungal peptidases in the epi- and mesopelagic realm and thus an active participation in oceanic protein degradation (Fig. 1 A, B). Interestingly, the fungal proteases (abundance and expression) were strongly related to the fungal CAZymes (Fig. 2). This indicates a close coupling between the degradation of carbohydrates and proteins by pelagic fungi in the oceanic water column. The abundance of both total and secretory peptidase genes and transcripts (and the percentage of secretory) was higher in the mesopelagic than in the upper water layers (SRF, MXL, DCM) (Fig. 1 A and B). The α-diversity of fungal protease genes and transcripts was also generally higher in the mesopelagic, with a slightly higher diversity in the micro-fraction (Fig. 1C). This increase with depth in total and secretory fungal proteases, their diversity, and expression was also observed for fungal CAZymes [12], again confirming the close linkage between the carbon and nitrogen cycling also in deep waters. Only secretory (but not total) prokaryotic proteases and CAZymes increase with water column depth [13], confirming the greater importance of secreted enzymes (both cell-associated and cell-free ones) in the degradation of organic matter with depth observed from both rate measurement [22, 23] and genomic approaches [13]. These results also imply that this increase with depth of secretory enzymes is a universal phenomenon for heterotrophs like heterotrophic bacteria and fungi in the ocean.

### Phylogenetic affiliation of genes and transcripts encoding fungal peptidases in the global ocean

**Fig. 3:**
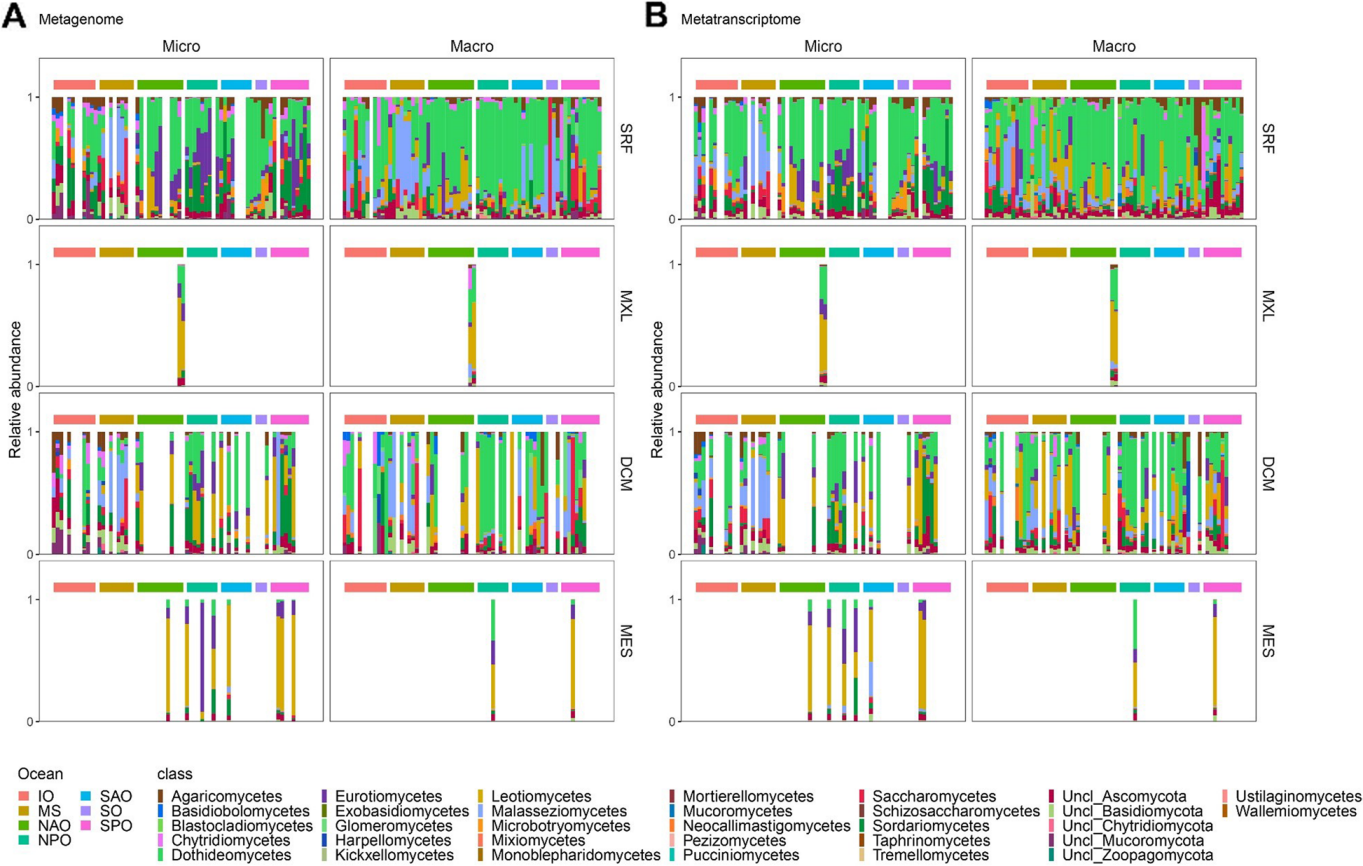
Phylogenetic affiliation of genes (**A**) and transcripts (**B**) encoding fungal peptidases at the class level. Micro, micro-mycobiome (0.8–5 um); macro, macro-mycobiome (5–2000 um). SRF, surface; MXL, mixed layer; DCM, deep chlorophyll maximum; MES, mesopelagic; IO, Indian Ocean; MS, Mediterranean Sea; NAO, North Atlantic Ocean; North Pacific Ocean; SAO, South Atlantic Ocean; SO, Southern Ocean; SPO, South Pacific Ocean

To reveal the main responsible fungal divisions for total (cytoplasmic and secreted) fungal peptidase activity, we analyzed the taxonomic affiliation of genes and transcripts. The genomic potential and expression patterns were similar, with Ascomycota dominating in most ocean basins and depths (Fig. S2). The relative abundance of peptidase genes and transcripts affiliated to Basidiomycota was higher in the Mediterranean Sea, the Indian Ocean, and the Southern Ocean than in the Pacific and Atlantic. On the contrary, the mesopelagic realm was dominated by Ascomycota. Mucoromycota and Chytridiomycota played a minor role in the overall abundance of fungal proteases and transcripts. The micro- and macro-mycobiome were dominated by proteases affiliated to the same phyla. Only Mucoromycota contributed slightly to fungal proteases in surface waters of the macro-size fraction in the South Pacific. These results are in accordance with the dominant phyla found to be responsible for the global expression of glucoside hydrolases (GH) [11] and of all CAZyme families [12] in pelagic fungi. Furthermore, our findings are consistent with a metagenomic study investigating the global phylogenetic and functional diversity of epi- and mesopelagic fungi [10]. This implies that the major part of the CAZyme and protease activity, and therefore the degradation of proteins and carbohydrates by mycoplankton in the oceans, is dominated by only two phyla (Ascomycota and Basidiomycota). Other phyla like Chytridiomycota, however, might become sporadically abundant and important by responding to phytoplankton bloom events [6, 24]. To gain deeper insights into the taxonomic affiliation of pelagic fungal proteases, we analyzed total (cytoplasmic and secreted) fungal peptidase genes and transcripts on fungal class level. Our results revealed that Dothideomycetes dominated the fungal protease pool in the surface layers and Leotiomycetes and Eurotiomycetes in the deep (Fig. 3). These classes were also found to dominate the abundance and expression of fungal CAZymes in the water column [12]. In contrast to the fungal CAZymes dynamics [12], some pronounced geographical fluctuations were found in the relative abundance of classes affiliated to fungal proteases, such as a relative increase in the contribution of Eurotiomycetes of the micro-fraction of the North Atlantic Ocean and in the North Pacific Ocean and of *Malassezia* mycota in the Mediterranean Sea. Interestingly, although the same classes dominated the abundance and expression of proteases and CAZymes in the water column, the diversity of fungal classes affiliated to the peptidases genes and transcripts was higher than for fungal CAZymes [12]. In summary, fungal proteases abundance and expression in all ocean basins and depths are dominated by the same fungal classes (Dothideomycetes in the surface and Leotiomycetes and Eurotiomycetes in the deep), thus representing major players in the fungal enzymatic cleavage of proteins and carbohydrates in the ocean.

### Functional classification of genes and transcripts encoding fungal peptidases in the global ocean

**Fig. 4:**
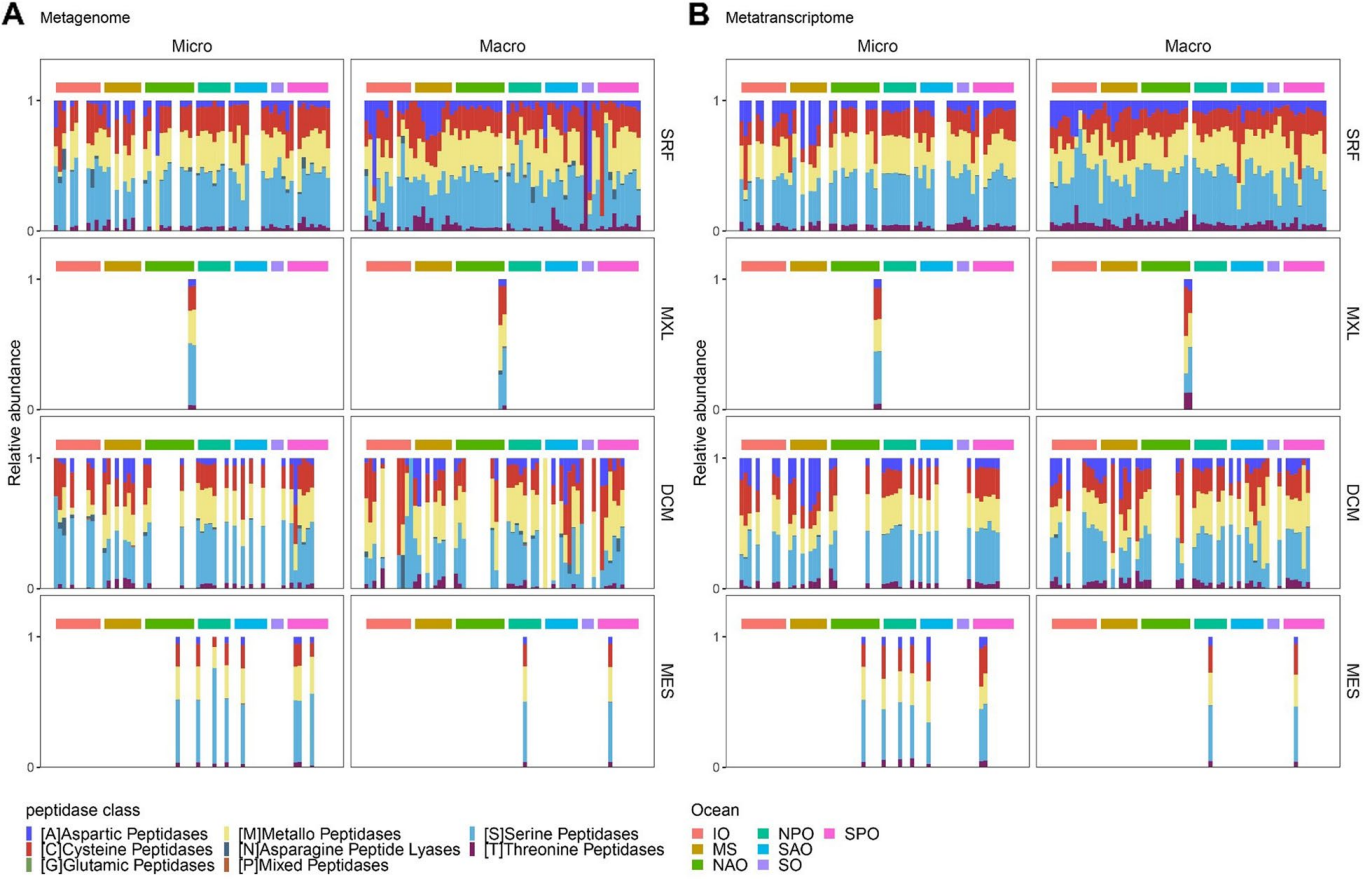
Functional classification of genes (**A**) and transcripts (**B**) encoding fungal peptidases. Micro, micro-mycobiome (0.8–5 μm); macro, macro-mycobiome (5–2000 μm). SRF, surface; MXL, mixed layer; DCM, deep chlorophyll maximum; MES, mesopelagic; IO, Indian Ocean; MS, Mediterranean Sea; NAO, North Atlantic Ocean; North Pacific Ocean; SAO, South Atlantic Ocean; SO, Southern Ocean; SPO, South Pacific Ocean

To decipher the functional roles of fungal peptidases in the water column, we investigated the distribution of genes and transcripts of peptidase classes in different ocean basins and depths. All ocean basins depths and size fractions were dominated by serine peptidases, followed by metallopeptidases, together contributing about 70% to the total genes and transcripts (Fig. 4). Similar observations were reported for the relative abundance of total prokaryotic peptidase genes and transcripts [13]. Additionally, other families represented were cysteine peptidases, followed by aspartic peptidases and threonine peptidases, with the latter two exhibiting a higher relative contribution in the MetaG of the surface macro-mycobiome of the Mediterranean Sea, the Southern Ocean, and the Indian Ocean. These regional variations in protease composition and dominance might be associated with the shift in dominance observed between Ascomycota to Basidiomycota as mentioned before for the same areas (Fig. S2). Additionally, the relative abundances of peptidase families were rather stable with depth, which contrasts the changes in the taxonomic affiliation with depth. Interestingly, the same depth pattern was reported for fungal CAZymes [12] and oceanic prokaryotic CAZymes and peptidases [13], pointing towards functional redundancy of fungi and prokaryotes in the degradation of carbohydrates and proteins in the oceanic water column.

### Taxonomic and functional affiliation of genes and transcripts encoding fungal secretory peptidases in the global ocean

As the abundance and relative percentage of secretory proteases increased with depth as also observed for fungal CAZymes [12], we additionally analyzed the taxonomic and functional affiliation of fungal secretory peptidases in the water column. Overall, the pattern of secretory peptidase genes and transcripts was very similar to that of the total peptidase genes, with Ascomycota dominating global oceans and becoming even more important with increasing depths (Fig. S3). Also, the taxonomic affiliation of fungal protease genes and transcripts at the class level was dominated by the same main groups responsible for total peptidase genes and transcripts (Fig. S4). Chytridiomycota do not seem to secrete peptidases, consistent with the findings on fungal secretory CAZymes [12]. Also, the major fungal peptidase classes dominating the secretory and the total pool were basically the same, with serine and metallopeptidases being the most abundant secreted peptidase class, followed by cysteine peptidases (which slightly increased in relative abundance in the secretory relative to the total pool) (Fig. S5). This is in contrast to oceanic prokaryotes, which secret only a small fraction of cysteine peptidases (according to the metatranscriptomics and metaproteomics analysis despite representing a major fraction in the metagenome) [13]. The contrasting composition of the secretory proteases between prokaryotes and fungi suggests different ecological strategies of these two main planktonic groups.

### Unique and shared protease pools between oceanic fungi and prokaryotes

To further investigate the potentially different roles fungi and prokaryotes play in the degradation of proteins in the ocean, we determined the number of unique and shared proteases between pelagic fungi and bacteria. After categorizing all marine bacterial and fungal proteases, we found that 81 oceanic fungal protease families were shared with bacteria, 4 were unique fungal proteases, and 108 were unique bacterial proteases (Table S1). The four unique fungal protease families included one metalloprotease, two serine proteases, and one cysteine protease, specifically tryptophanyl aminopeptidase (M77), nucleoporin 145 (S59), Ssy5 peptidase (S64), and otubain-1 (C65). Most of the existing fungal protease data are derived from *Saccharomyces cerevisiae* (Saccharomycotina) or other fungal taxa. Hence, these functional data have to be interpreted with caution. The conclusion of our analyses of these four unique fungal proteases appears, however, consistent with the available literature. Tryptophanyl aminopeptidase has been reported to originate from a common fungal ancestor and hence can only be found in the kingdom of fungi [25]. Also, Ssy5 peptidase, involved in sensing of extracellular amino acids and in defense mechanisms against viruses, is supposed to be a fungal evolutionary innovation, which originates from Dikarya or was lost during later evolution [26]. Thus, it is reasonable that tryptophanyl aminopeptidase and Ssy5 peptidase were exclusively found in the fungal fraction. Nucleoporin 145, which is essential for nuclear pore formation [27], is shared between plants, animals, SAR (stramenopiles, alveolates, Rhizaria), and fungi [26]. Otubain-1 is a common eukaryotic isopeptidase involved in ubiquitin-based cell-signaling mechanisms [28] and absent in oceanic bacteria. Also, both Nucleoporin 145 and Otubain-1 were found to be unique to eukaryotic peptidase families [29].

### Global abundance and expression of the dominant fungal protease families and subfamilies in the ocean

**Fig. 5:**
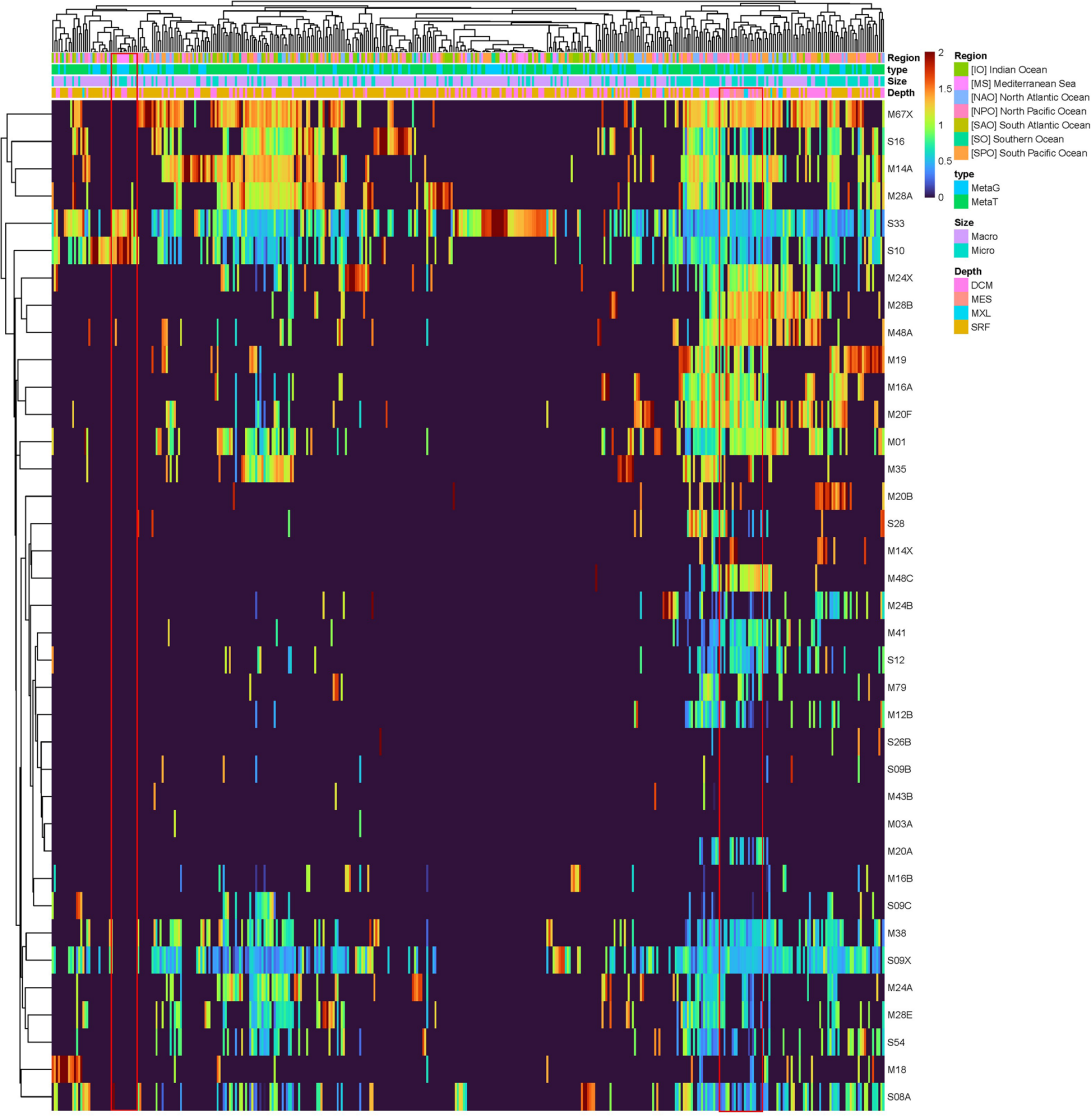
Heatmap of fungal serine and metallo subfamily peptidase profile with abundance > 1%. Red frames highlight peptidase actively expressed in the Mediterranean Sea and the mesopelagic zone. Micro, micro-mycobiome (0.8–5 um); macro, macro-mycobiome (5–2000 um). SRF, surface; MXL, mixed layer; DCM, deep chlorophyll maximum; MES, mesopelagic; IO, Indian Ocean; MS, Mediterranean Sea; NAO, North Atlantic Ocean; North Pacific Ocean; SAO, South Atlantic Ocean; SO, Southern Ocean; SPO, South Pacific Ocean; MetaG, metagenomic; MetaT, metatranscriptomic

To identify the main functions involved in the fungal degradation of proteins in the ocean, we determined the abundance and expression of the main subfamilies of serine and metallopeptidases (the dominant pelagic fungal protease families) (Fig. 5). However, some subfamilies including “X” in their name denote inactive peptidases, despite their occasionally widespread expression (e.g., S09X, Fig. 5). Nevertheless, a distinct clustering pattern was found in mesopelagic waters with a relatively high expression of a variety of subfamilies and relatively more metallopeptidases than serine peptidases compared to shallower layers. Also, some subfamilies such as glutamate carboxypeptidase (M20A), Oma1 peptidase (M48C), FtsH peptidase (M41), and D-Ala-D-Ala carboxypeptidase B (S12) were almost exclusively found to be expressed in deep waters. M20A is responsible for cleaving vitamin B9 to retrieve pteroate and L-glutamate [30], which might counteract the normally scarce availability of glutamate in deep waters [31]. D-Ala-D-Ala carboxypeptidase B (S12) is involved in chitinase degradation [32]. Transcript-based analyses (Fig. 5) allowed deciphering one distinct protease cluster from the Mediterranean Sea exhibiting a lower diversity of transcripts compared to other ocean basins and only expressing prolyl aminopeptidase (S33) and carboxypeptidase Y (S10) (Fig. 5). It is worth mentioning that S33 and S10 are the most widely expressed proteases globally. S33 has recently been suggested as a marker for sapotrophy in fungi as it is assumed to be less expressed in fungi with a pathogenic or animal-associated lifestyle [26]. However, S33 also includes some abhydrolases, which additionally degrade other substrates like lipids. S10 is a vacuole protease [33] and also involved in extracellular organic matter degradation [34]. The global dominance of and co-occurrence of S10 (plant associated) and S33 (less expressed in fungal pathogens) might indicate that the majority of the oceanic mycobiome is dominated by saprotrophy rather than pathogeny. This assumption is further supported by the relatively high expression of the aminopeptidases M01, M24, M28, and S12, which are all indicative of a saprotrophic nutrition. Additionally, subtilisin Carlsberg (S08A), a subfamily reported to be involved in nutrition and mostly secreted from the cell [16], was widely expressed. Since our analyses indicated that Chytridiomycota do not contribute to the secretory pool of proteases (Fig. S3), this suggests fungi belonging to the subkingdom Dikarya as the main producers of S08A. This might imply that besides their role as parasites and associated processes described as the fungal shunt and mycoloop [7, 9], fungi are active contributors to the organic matter cycling in the ocean and hence play a major role in the biogeochemical cycles in the global ocean.

## Conclusions

**Fig. 6:**
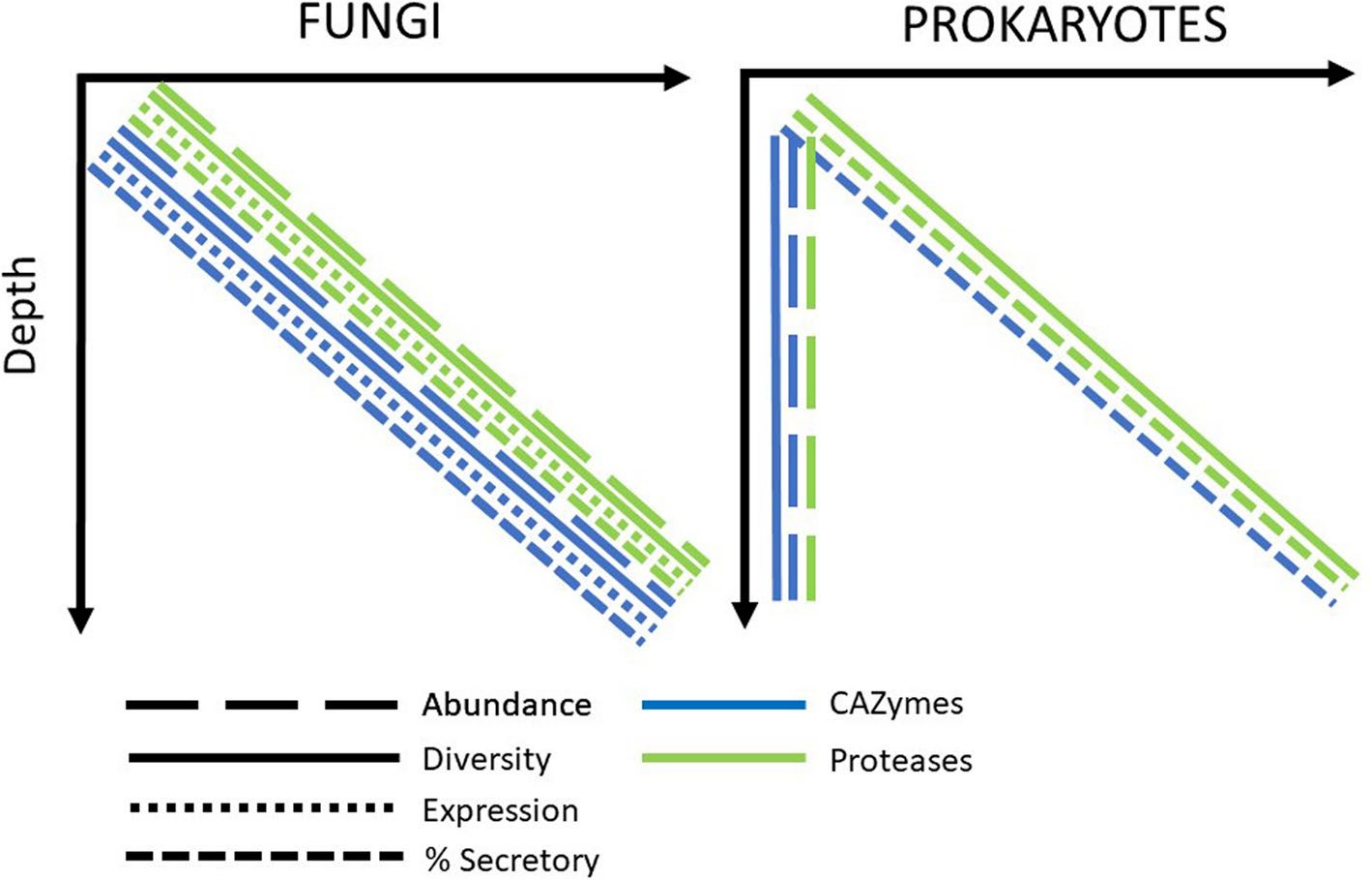
Diagram depicting the depth-related patterns in the total gene abundance, expression, diversity, and the percentage of secretory peptidases and CAZymes from pelagic fungi and prokaryotes. The pattern associated with fungal proteases are derived from the present study, the  fungal CAZymes patterns originates from Baltar et al. [12], and the prokaryotic CAZymes and peptidases trends are derived from Zhao et al. [13]

Our findings reveal that fungi are globally active contributors to oceanic protein degradation and thus key players in the nitrogen cycling in the water column, especially in deeper waters. This increasing abundance, diversity, and expression of total and secretory fungal proteases with water column depth is consistent with the distribution pattern of fungal CAZymes [12] and might be related to the degradation of more refractory organic material in the dark ocean and/or the sinking of particles. In contrast, the total proteases and CAZymes of prokaryotes do not increase with depth [13], indicating a potentially different contribution or role of prokaryotes versus fungi in the deep waters (Fig. 6). The percentage of secretory fungal proteases also increased with depth, similar to fungal CAZymes [12] and prokaryotic proteases and CAZymes [13] (Fig. 6). This highlights the key role of secreted enzymes in the degradation of organic matter [23], probably indicative of a preferential particle-attached lifestyle [13]. This also implies that the increasing fraction of secretory enzymes with depth is a conserved phenomenon across pelagic prokaryotes and fungi. However, pronounced differences were found in the composition of secretory peptidases between prokaryotes and fungi, indicating different strategies in the use of secretory peptidase between prokaryotes and fungi in the ocean. We also identified the main taxa and functions affiliated to fungal proteases, revealing Ascomycota (dominated by Dothideomycetes in epipelagic and Leotiomycetes and Eurotiomycetes in mesopelagic) and Basidiomycota as the main responsible for fungal protease expression in all depths and ocean basins. Chytridiomycota were only regionally important (probably due to their more temporal “blooming” lifestyle). These Ascomycota and Basidiomycota are also the taxa dominating the fungal CAZyme pool [12], indicating that those are the major players in the fungal enzymatic cleavage of both proteins and carbohydrates in the ocean. Most of the protease families used by oceanic fungi are shared with prokaryotes, although 4 unique proteases were detected in fungi. The most widely used oceanic fungal peptidases classes were serine, followed by metalloproteases. The expression profile of protease subfamilies suggests that the majority of the pelagic fungal communities is dominated by saprotrophy rather than parasitism. The composition of the main protease classes was conserved with depth, irrespective of shifts in the taxonomic affiliation, which indicates functional redundancy in fungal communities. Interestingly, functional redundancy with depth was also reported for fungal CAZymes [12] and oceanic prokaryotic CAZymes and peptidases [13], indicating that functional redundancy might be a common trait of pelagic fungal and prokaryotic communities in the degradation of organic matter (at least for carbohydrates and proteins) in the oceanic water column. Collectively, this study highlights the active contribution of fungi in the degradation of proteins, which are the major macromolecular compound class in living and detrital biomass in the ocean. Furthermore, our results also point towards contrasting strategies in the enzymatic degradation of organic matter by fungi and prokaryotes in the global ocean.

## Supplementary Information

**Additional file 1: Figure S1.** Principal coordinate analysis of fungal peptidase fungal genes present in the metagenome (A) and in the metatranscriptome (B) and environmental drivers shaping fungal peptidase gene composition at both metagenomic and metatranscriptomic level (C). Micro, micro-mycobiome (0.8-5 μm); Macro, macro-mycobiome (5-2,000 μm). SRF, surface; MXL, mixed layer; DCM, deep chlorophyll maximum; MES, mesopelagic. **Figure S2.** Phylogenetic affiliation of genes (A) and transcripts (B) encoding fungal peptidases at the phylum level. Micro, micro-mycobiome (0.8-5 μm); Macro, macro-mycobiome (5-2000 μm). SRF, surface; MXL, mixed layer; DCM, deep chlorophyll maximum; MES, mesopelagic; IO, Indian Ocean; MS, Mediterranean Sea; NAO, North Atlantic Ocean; North Pacific Ocean; SAO, South Atlantic Ocean; SO, Southern Ocean; SPO, South Pacific Ocean. **Figure S3.** Phylogenetic affiliation of genes (A) and transcripts (B) encoding secretory fungal peptidases at the phylum level. Micro, micro-mycobiome (0.8-5 μm); Macro, macro-mycobiome (5-2000 μm). SRF, surface; MXL, mixed layer; DCM, deep chlorophyll maximum; MES, mesopelagic; IO, Indian Ocean; MS, Mediterranean Sea; NAO, North Atlantic Ocean; North Pacific Ocean; SAO, South Atlantic Ocean; SO, Southern Ocean; SPO, South Pacific Ocean. **Figure S4.** Phylogenetic affiliation of genes (A) and transcripts (B) encoding secretory fungal peptidases at the class level. Micro, micro-mycobiome (0.8-5 μm); Macro, macro-mycobiome (5-2000 μm). SRF, surface; MXL, mixed layer; DCM, deep chlorophyll maximum; MES, mesopelagic; IO, Indian Ocean; MS, Mediterranean Sea; NAO, North Atlantic Ocean; North Pacific Ocean; SAO, South Atlantic Ocean; SO, Southern Ocean; SPO, South Pacific Ocean. **Figure S5.** Functional classification of genes (A) and transcripts (B) encoding secretory fungal peptidases. Micro, micro-mycobiome (0.8-5 μm); Macro, macro-mycobiome (5-2000 μm). SRF, surface; MXL, mixed layer; DCM, deep chlorophyll maximum; MES, mesopelagic; IO, Indian Ocean; MS, Mediterranean Sea; NAO, North Atlantic Ocean; North Pacific Ocean; SAO, South Atlantic Ocean; SO, Southern Ocean; SPO, South Pacific Ocean.

**Additional file 2: Table S1.** Families of proteases affiliated to Fungi, Bacteria and shared by both.

**Additional file 3.** Dataset S1.

## Data Availability

All data used in this work are publicly available. The sequences and occurrences in the corresponding metagenomes and metatranscriptomes of marine eukaryotic genes were downloaded from Carradec et al., a global ocean atlas of eukaryotic genes (*Nature Communications* 9, 373, doi:10.1038/s41467-017-02342-1 (2018)). Additionally, we provide an annotation dataset including annotation table and fungal peptidase sequences (Supplementary Dataset S1).
