# Supplementary material for: Global contribution of pelagic fungi to protein degradation in the ocean
Source: Microbiome. 2022 Sep 1;10:143. doi: 10.1186/s40168-022-01329-5 (PMC9434897; doi:10.1186/s40168-022-01329-5)
Supplement: Supplementary file 2 — Additional file 1: Figure S1. Principal coordinate analysis of fungal peptidase fungal genes present in the metagenome (A) and in the metatranscriptome (B) and environmental drivers shaping fungal peptidase gene composition at both metagenomic and metatranscriptomic level (C). Micro, micro-mycobiome (0.8-5 μm); Macro, macro-mycobiome (5-2,000 μm). SRF, surface; MXL, mixed layer; DCM, deep chlorophyll maximum; MES, mesopelagic. Figure S2. Phylogenetic affiliation of genes (A) and transcripts (B) encoding fungal peptidases at the phylum level. Micro, micro-mycobiome (0.8-5 μm); Macro, macro-mycobiome (5-2000 μm). SRF, surface; MXL, mixed layer; DCM, deep chlorophyll maximum; MES, mesopelagic; IO, Indian Ocean; MS, Mediterranean Sea; NAO, North Atlantic Ocean; North Pacific Ocean; SAO, South Atlantic Ocean; SO, Southern Ocean; SPO, South Pacific Ocean. Figure S3. Phylogenetic affiliation of genes (A) and transcripts (B) encoding secretory fungal peptidases at the phylum level. Micro, micro-mycobiome (0.8-5 μm); Macro, macro-mycobiome (5-2000 μm). SRF, surface; MXL, mixed layer; DCM, deep chlorophyll maximum; MES, mesopelagic; IO, Indian Ocean; MS, Mediterranean Sea; NAO, North Atlantic Ocean; North Pacific Ocean; SAO, South Atlantic Ocean; SO, Southern Ocean; SPO, South Pacific Ocean. Figure S4. Phylogenetic affiliation of genes (A) and transcripts (B) encoding secretory fungal peptidases at the class level. Micro, micro-mycobiome (0.8-5 μm); Macro, macro-mycobiome (5-2000 μm). SRF, surface; MXL, mixed layer; DCM, deep chlorophyll maximum; MES, mesopelagic; IO, Indian Ocean; MS, Mediterranean Sea; NAO, North Atlantic Ocean; North Pacific Ocean; SAO, South Atlantic Ocean; SO, Southern Ocean; SPO, South Pacific Ocean. Figure S5. Functional classification of genes (A) and transcripts (B) encoding secretory fungal peptidases. Micro, micro-mycobiome (0.8-5 μm); Macro, macro-mycobiome (5-2000 μm). SRF, surface; MXL, mixed layer; DCM, deep chlorophyll maximum; MES, mesopelagi [file 40168_2022_1329_MOESM1_ESM.docx]

**Supplementary Information for**

*Global contribution of pelagic fungi to protein degradation in the oceans*

Eva Breyer^1*#^, Zihao Zhao^1*#^, Gerhard J. Herndl^1,2,3^, Federico Baltar^1^

^1^Department of Functional and Evolutionary Ecology, University of Vienna, Djerassiplatz 1, 1030 Vienna, Austria

^2^NIOZ, Department of Marine Microbiology and Biogeochemistry, Royal Netherlands Institute for Sea Research, Utrecht University, AB Den Burg, The Netherlands

^3^Vienna Metabolomics Center, University of Vienna, Althanstraße 14, A-1090 Vienna, Austria

* Correspondence: Eva, Breyer, Zihao Zhao, Federico Baltar

^#^ Shared first co-authorship

**Email:**  eva.breyer@univie.ac.at, [zihao.zhao@univie.ac.at](mailto:xxxxx@xxxx.xxx)

**This PDF file includes:**

Ext. Figures S1-S5

**Other supplementary materials for this manuscript include the following:**

Table S1

Dataset S1


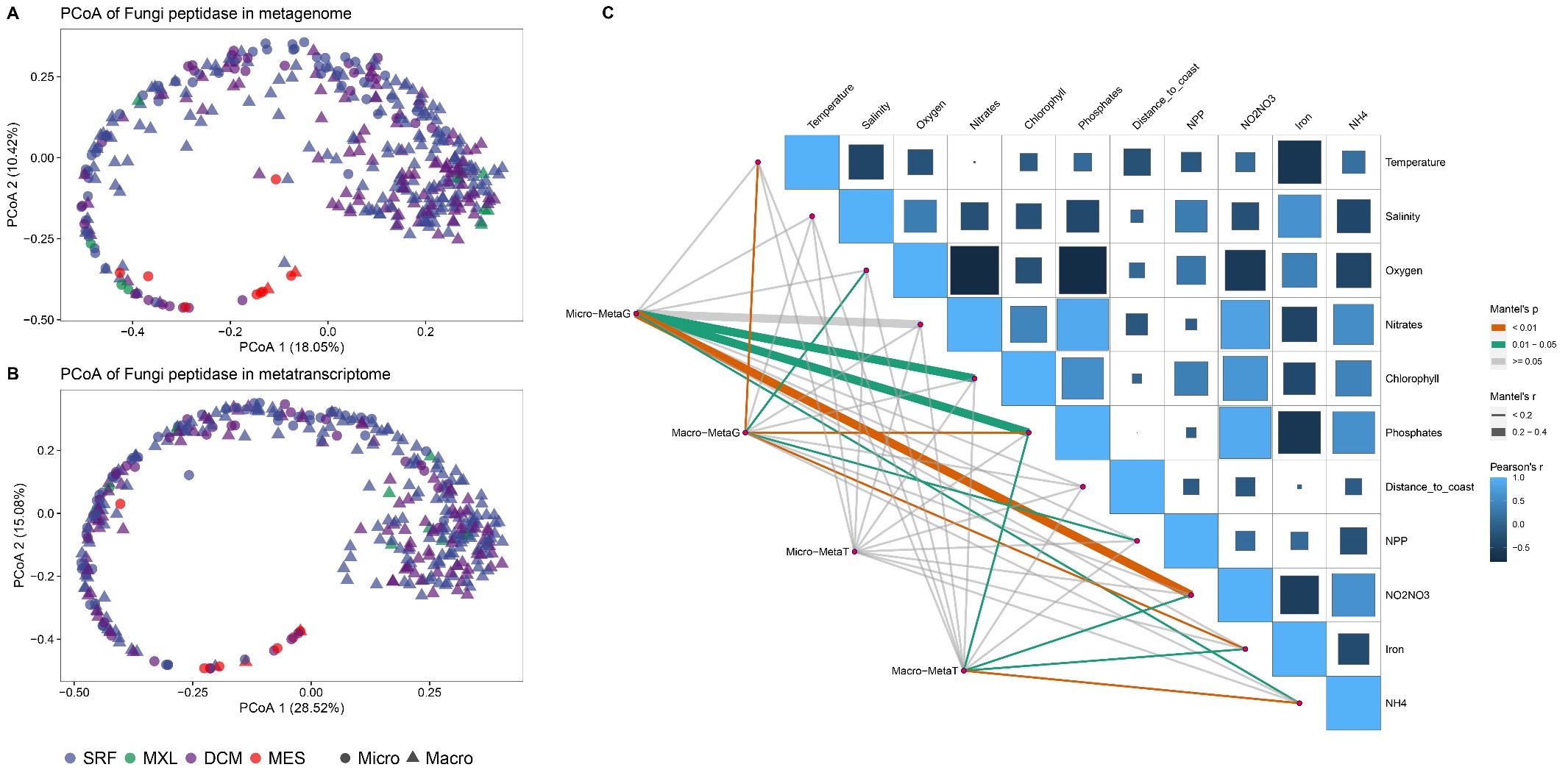


**Fig. S1** Principal coordinate analysis of fungal peptidase fungal genes present in the metagenome (A) and in the metatranscriptome (B) and environmental drivers shaping fungal peptidase gene composition at both metagenomic and metatranscriptomic level (C). Micro, micro-mycobiome (0.8-5 µm); Macro, macro-mycobiome (5-2,000 µm). SRF, surface; MXL, mixed layer; DCM, deep chlorophyll maximum; MES, mesopelagic.


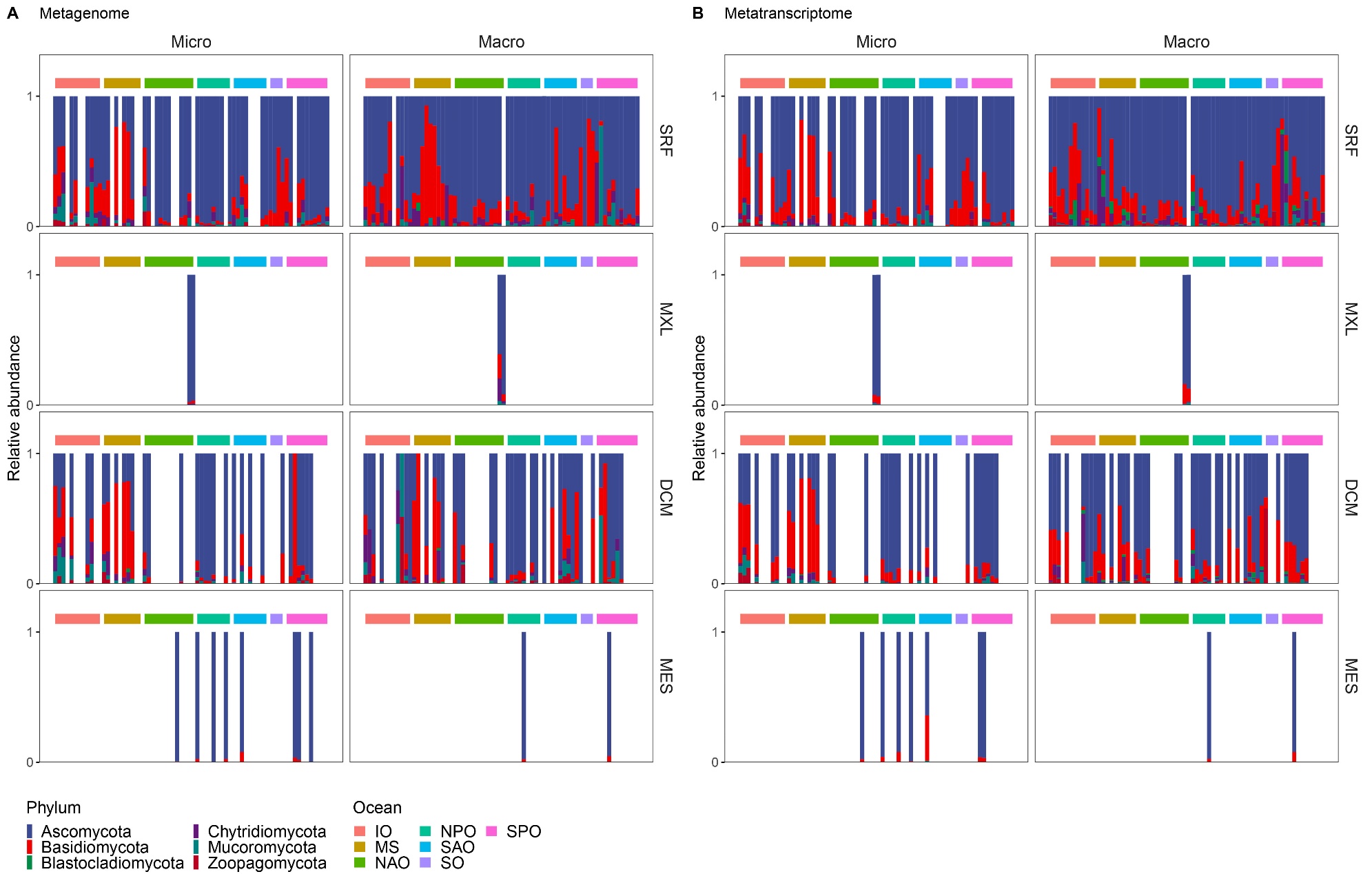


**Fig. S2** Phylogenetic affiliation of genes (A) and transcripts (B) encoding fungal peptidases at the phylum level. Micro, micro-mycobiome (0.8-5 µm); Macro, macro-mycobiome (5-2000 µm). SRF, surface; MXL, mixed layer; DCM, deep chlorophyll maximum; MES, mesopelagic; IO, Indian Ocean; MS, Mediterranean Sea; NAO, North Atlantic Ocean; North Pacific Ocean; SAO, South Atlantic Ocean; SO, Southern Ocean; SPO, South Pacific Ocean.


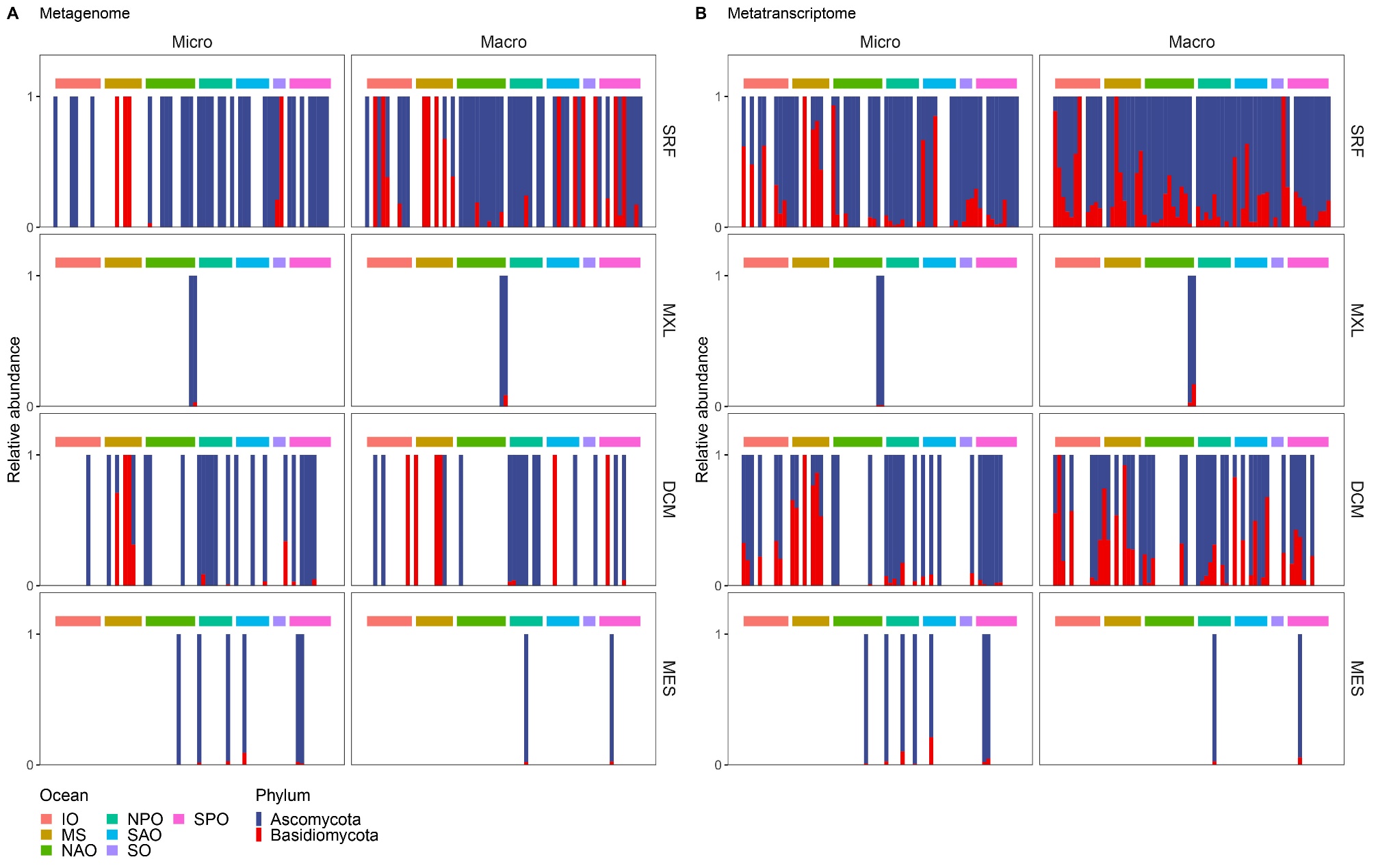


**Fig. S3** Phylogenetic affiliation of genes (A) and transcripts (B) encoding secretory fungal peptidases at the phylum level. Micro, micro-mycobiome (0.8-5 µm); Macro, macro-mycobiome (5-2000 µm). SRF, surface; MXL, mixed layer; DCM, deep chlorophyll maximum; MES, mesopelagic; IO, Indian Ocean; MS, Mediterranean Sea; NAO, North Atlantic Ocean; North Pacific Ocean; SAO, South Atlantic Ocean; SO, Southern Ocean; SPO, South Pacific Ocean.


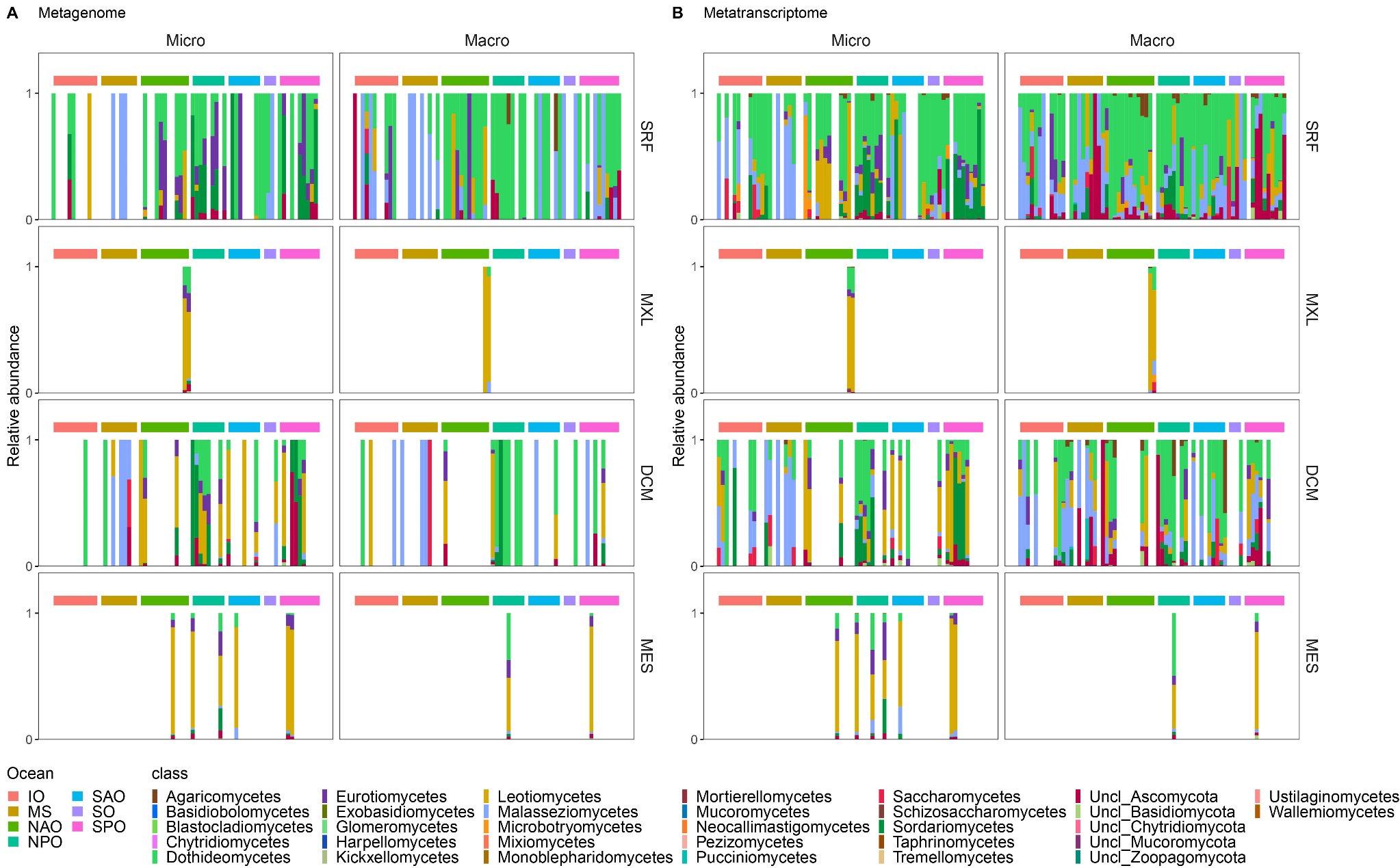


**Fig. S4** Phylogenetic affiliation of genes (A) and transcripts (B) encoding secretory fungal peptidases at the class level. Micro, micro-mycobiome (0.8-5 µm); Macro, macro-mycobiome (5-2000 µm). SRF, surface; MXL, mixed layer; DCM, deep chlorophyll maximum; MES, mesopelagic; IO, Indian Ocean; MS, Mediterranean Sea; NAO, North Atlantic Ocean; North Pacific Ocean; SAO, South Atlantic Ocean; SO, Southern Ocean; SPO, South Pacific Ocean.


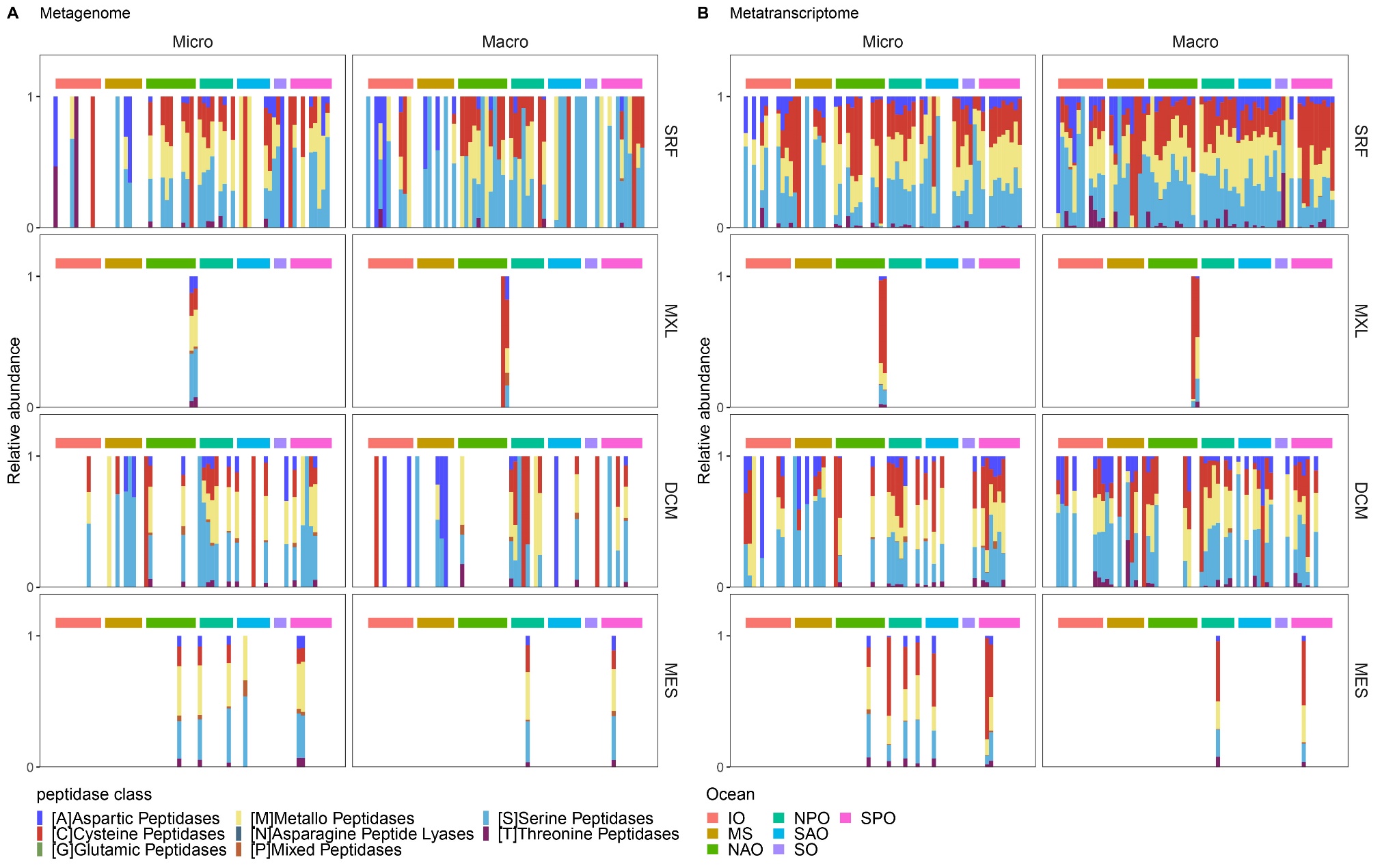


**Fig. S5** Functional classification of genes (A) and transcripts (B) encoding secretory fungal peptidases. Micro, micro-mycobiome (0.8-5 µm); Macro, macro-mycobiome (5-2000 µm). SRF, surface; MXL, mixed layer; DCM, deep chlorophyll maximum; MES, mesopelagic; IO, Indian Ocean; MS, Mediterranean Sea; NAO, North Atlantic Ocean; North Pacific Ocean; SAO, South Atlantic Ocean; SO, Southern Ocean; SPO, South Pacific Ocean.
